# Correction: Yu et al. The Role of circTmeff-1 in Morphine Addiction Memory of Mice. *Cells* 2023, *12*, 1985

**DOI:** 10.3390/cells13242129

**Published:** 2024-12-23

**Authors:** Hailei Yu, Boyang Wen, Yun Lu, Bing Xie, Feng Yu, Minglong Zhang, Chunling Ma, Bin Cong, Di Wen, Haitao Bi

**Affiliations:** 1 Hebei Key Laboratory of Forensic Medicine, Collaborative Innovation Center of Forensic Medical Molecular Identification, Research Unit of Digestive Tract Microecosystem Pharmacology and Toxicology, College of Forensic Medicine, Hebei Medical University, Chinese Academy of Medical Sciences, Shijiazhuang 050000, China; 18232587910@163.com (H.Y.); boyangwen@126.com (B.W.); 15226523170@163.com (Y.L.); queenie06@163.com (B.X.); fengyu1405@163.com (F.Y.); chunlingma@126.com (C.M.); hbydbincong@126.com (B.C.); 2 Department of Biogenetics, Qiqihar Medical University, Qiqihar 161000, China; jylinyao@163.com

In the original publication [1], the funder “the Graduate Innovation Funding Project of Hebei Medical University, XCXZZS202302” to Boyang Wen was not included. The correct Funding appears below.

**Funding:** This research was funded by the Natural Science Foundation of Hebei Province (grant number H2022206085), the National Natural Science Foundation of China (grant number 81871524), Postdoctoral Research Project of Hebei Province (grant number 2022005010), and Youth Foundation of Science and Technology Research Project of colleges and Universities of Hebei Province (grant number QN2023219), and the Graduate Innovation Funding Project of Hebei Medical University, (grant number XCXZZS202302).

There was another mistake in Figure 3 as published. The two charts in Figure 3Bb are duplicated. The corrected Figure 3 appears below.

The authors state that the scientific conclusions are unaffected. This correction was approved by the Academic Editor. The original publication has also been updated.

## Figures and Tables

**Figure 3:**
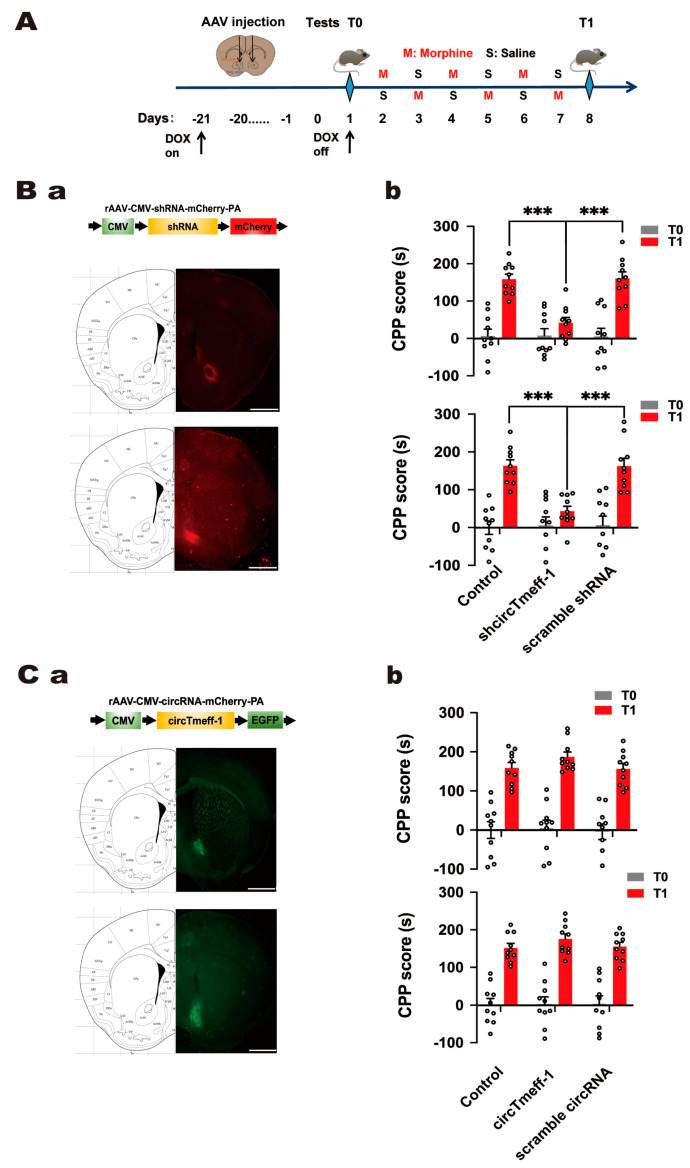
CircTmeff-1 modulates background-induced memory formation of morphine addiction. (**A**): a schematic diagram of the timeline of the morphine CPP training program. (**B**): Reducing circTmeff-1 expression in the NAc inhibits the expression of morphine addiction memory. (**a**) Schematic diagram of AAV structure used to down-regulate circTmeff-1; mCherry fluorescence location indicated that the injected virus was located in the core or shell of the NAc; scale bar = 1000 µm. (**b**) Down-regulation of circTmeff-1 in both NAc core and shell inhibited morphine seeking (*n* = 10 per group), *** *p* < 0.001. (**C**): Overexpression of circTmeff-1 in the core and shell of the NAc did not affect the expression of morphine-addicted memory. (**a**) Schematic diagram of AAV structure used for overexpression of circTmeff-1; EGFP fluorescence location indicated that the injected virus was located in the core or shell of NAc; scale bar = 1000 µm. (**b**) Overexpression of circTmeff-1 in the core and shell of the NAc had no effect on T1 background-induced morphine seeking (*n* = 10 per group). EGFP, enhanced green fluorescent protein.
